# Analysis of *MUC6* Genetic Variants on the Clinicopathologic Characteristics of Patients with Hepatocellular Carcinoma

**DOI:** 10.7150/jca.75754

**Published:** 2022-09-06

**Authors:** Hsiang-Lin Lee, Yi-Chung Chien, Hsiang-Ling Wang, Chun-Hung Hua, Liang-Chih Liu, Guo-Wei Wu, Li-Yuan Bai, Shun-Fa Yang, Yung-Luen Yu

**Affiliations:** 1 School of Medicine, Chung Shan Medical University, Taichung 40201, Taiwan.; 2 Institute of Medicine, Chung Shan Medical University, Taichung 40201, Taiwan.; 3 Department of Surgery, Chung Shan Medical University Hospital, Taichung 40201, Taiwan.; 4 Graduate Institute of Biomedical Sciences, China Medical University, Taichung 40402, Taiwan.; 5 Ph.D. Program for Translational Medicine, China Medical University, Taichung 40402, Taiwan.; 6 Institute of Translational Medicine and New Drug Development, Taichung 40402, Taiwan.; 7 Drug Development Center, Research Center for Cancer Biology, China Medical University, Taichung 40402, Taiwan.; 8 Center for Molecular Medicine, China Medical University Hospital, Taichung 40402, Taiwan.; 9 Department of Beauty Science, National Taichung University of Science and Technology, Taichung 40404, Taiwan.; 10 Department of Otorhinolaryngology Head and Neck Surgery, China Medical University Hospital, Taichung, Taiwan.; 11 School of Medicine, College of Medicine, China Medical University, Taichung 40402, Taiwan.; 12 Department of Surgery, China Medical University Hospital, Taichung 40402, Taiwan.; 13 Department of Hematology and Oncology, China Medical University Hospital, Taichung 40402, Taiwan.; 14 Institute of Medicine, Chung Shan Medical University, Taichung 40201, Taiwan.; 15 Department of Medical Research, Chung Shan Medical University Hospital, Taichung 40201, Taiwan.; 16 Department of Medical Laboratory Science and Biotechnology, Asia University, Taichung 41354, Taiwan.

**Keywords:** hepatocellular carcinoma, *MUC6*, Child-Pugh score, single-nucleotide polymorphisms

## Abstract

Hepatocellular carcinoma (HCC) is the leading malignancy associated with cancer-related deaths worldwide. Many studies have indicated that mucin (MUC) expression plays an important role in cancer metastasis and recurrence. *MUC6* expression is observed in gastric and oncocytic phenotypes and may play an important role during cancer progression. We found the level of *MUC6* is lower in HCC patients but did not affect the survival of HCC patients. Therefore, in this study, we investigated the combined effect of *MUC6* polymorphisms and exposure to environmental carcinogens on the susceptibility to and clinicopathological characteristics of HCC. Three single-nucleotide polymorphisms (SNPs) of *MUC6* (rs61869016, rs6597947, and rs7481521) from 1197 healthy controls and 423 HCC patients were analyzed using real-time PCR. After adjusting for other co-variants, we found that carrying a CC genotype at *MUC6* rs61869016 had a lower risk of developing HCC than wildtype carriers. Moreover, patients with a smoking habit who carried the C allele of rs61869016 and T allele of rs7481521 had a higher (B or C) Child-Pugh score than other genotypes, suggesting significant functional compromise and decompensated disease. Therefore, our findings suggest that genetic variations in *MUC6* may corelate to HCC and indicate progression in HCC patients.

## Introduction

The main risk factors for liver cancer are hepatitis virus infection and cirrhosis, as well as chronic hepatitis, which leads to cirrhosis and then to liver cancer. Most of the symptoms of cirrhosis are the result of the progression of viral hepatitis, drug-related hepatitis, and alcoholic hepatitis. Mucin (MUC) is the main component of any mucus secretion, providing the mucus with its biophysiochemical properties as a function of its characteristics and degree of glycosylation 1, 2. Mucins play a role in both physiological and pathological conditions 3-7. Aberrant expression of mucins can lead to loss of epithelial cell polarity and promote epithelial-mesenchymal transition (EMT), which leads to increased cell motility and invasion, a critically important step in tumorigenesis 3, 8, 9.

It is generally accepted that hepatocellular carcinoma (HCC) does not produce mucins, whereas cholangiocarcinoma (CC) or combined/mixed hepatocellular cholangiocarcinoma (cHCC-CC) may produce these glycoproteins 10, 11. However, a growing number of reports have indicated that HCC cells that do not exhibit or that have not yet morphologically differentiated into the biliary phenotype can also produce mucins 12-15. Mucin 6 (MUC6) is one of the main components of the mucus barrier in the stomach, and it is secreted by the pyloric gland cells of the gastric sinus and the mucus neck cells located in the lower layer of the gastric mucosa. *MUC6* expression is observed in both gastric and cancer cell phenotypes. It has been reported that methylation of the *MUC6* promoter may lead to significant downregulation of *MUC6* in gastric cancer and promote the progression of gastric cancer 16. Furthermore, high *MUC6* expression is a characteristic in chronic viral hepatitis, which may induce hepatocellular carcinoma 17. However, the detailed role of the tissue expression of mucins in HCC tumor cells is not well understood.

A number of studies have reported genetic susceptibility factors that may be involved in HCC. For example, single-nucleotide polymorphisms (SNPs) are the most common type of DNA sequence variation that have shown the potential to predict cancer risk 18, 19. The expression of proteins or their functions may be altered by their SNPs, thus influencing the progression of cancer. The relationship between the expression of *MUC6* SNPs and chronic atrophic gastritis was revealed 20. However, the exact role of *MUC6* SNPs in cancer progression and development in Taiwanese HCC patients remains poorly investigated. In the current study, we selected three *MUC6* SNPs (rs61869016 (5'-UTR), rs6597947 (5'-UTR), and rs7481521 (exon)) with the aim of elucidating their correlations to Taiwanese HCC patients and cancer prognosis.

## Materials and Methods

### Study Participants and Specimen Collection

In this study, 423 HCC patients were recruited from Chung Shan Medical University Hospital in Taichung, Taiwan. All participants provided informed written consent during the registration process. HCC patients were clinically staged at the time of diagnosis according to the tumor/node/metastasis staging system of the American Joint Committee on Cancer (AJCC, 2002). The diagnosis of cirrhosis is based on liver biopsy or abdominal ultrasound. Clinical features, including liver cirrhosis, aspartate aminotransferase (AST), the levels of α-fetoprotein (AFP), alanine aminotransferase (ALT), tumor staging, tumor size, lymph-node metastasis, distant metastasis, presence of HBV surface antigen (HBsAg), and reactivity with antibody against HCV (anti-HCV), were collected from chart reviews. For the control group, 1197 individuals, between 20 and 70 years of age with no history of cancer, were selected from the Taiwan Biobank (https://www.twbiobank.org.tw).

The information on gender, age, cigarette smoking status, and alcohol drinking status was collected from each subject. An average of more than two drinks per day was considered alcohol consumption. Smoking of at least one cigarette per day in the latest 3 months was considered a persistent smoking habit. The research was approved by the Institutional Review Board of Chung Shan Medical University Hospital.

### Comprehensive Analysis of *MUC6* from The Cancer Genome Atlas (TCGA)

UALCAN is a comprehensive, user-friendly, and interactive web resource for analyzing cancer omics data (http://ualcan.path.uab.edu/index.html). UALCAN uses TCGA level 3 RNA-seq and clinical data from 31 cancer types 21. Gene expression profile interactive analysis 2 (GEPIA2, http://gepia2.cancer-pku.cn/#index) is a updated version of GEPIA for analyzing the RNA sequencing expression data of 9,736 tumors and 8,587 normal samples from the TCGA and the GTEx projects, using a standard processing pipeline 22. In this study, we used UALCAN and GEPIA2 for tumor/normal differential expression analysis and overall survial of *MUC6* expression in HCC patients.

### Selection of *MUC6* Polymorphisms

A total of three SNPs in *MUC6* (NM_005961.3) were selected from the International HapMap Project data for this study. We included the SNPs rs61869016 (5'-UTR), rs6597947 (5'-UTR), and rs7481521 (exon) of MUC6.

### *MUC6* Genotyping

Allelic discrimination of the *MUC6* polymorphisms rs61869016, rs6597947, and rs7481521 was assessed using an ABI StepOne real-time polymerase chain reaction system (Applied Biosystems), SDS v3.0 software (Applied Biosystems), and the TaqMan assay 18.

### Statistical Analyses

To evaluate the differences in age and demographic characteristics between the control groups and HCC patients, the Mann-Whitney U test was used. The odds ratios with 95% confidence intervals (CIs) were estimated using logistic regression models. A *p-*value <0.05 was considered significant. The data were analyzed using SAS statistical software.

## Results

**Figure 1:**
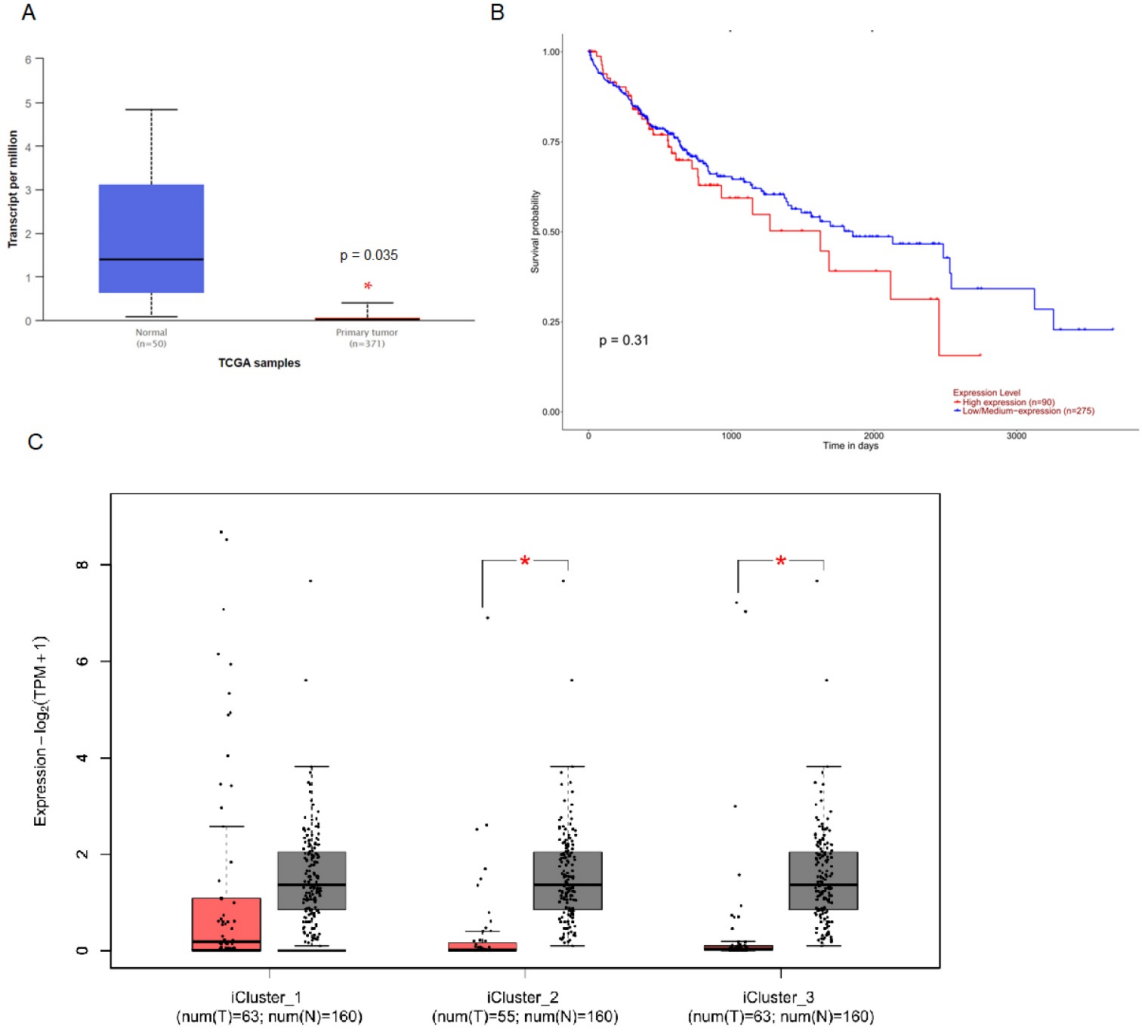
** The level of *MUC6* is correlated with HCC progression but not with the survival rate of HCC. (A)** The level of* MUC6* in normal control and hepatocellular carcinoma patients. **(B)** The overall survival of different levels of *MUC6* in HCC patients as assessed with data from UALCAN. **(C)** The level of *MUC6* in different subtypes of HCC patients. * *p* < 0.05.

To investigate the clinical impact of MUC6 on HCC progression, we used UALCAN and GEPIA 2 to assess the relationship between cellular levels of *MUC6* of normal people and HCC patients and the overall survival of HCC patients. The results indicated that the level of *MUC6* in normal people was significantly much higher than in all and different subtypes HCC patients (Figure 1A and 1C). Interestingly, the expression of MUC6 did not affect the overall survival of HCC patients. This result implies that the regulation of *MUC6* in HCC may have unknown mechanisms.

To identify possible factors causing HCC in clinical practice, a total of 1197 healthy controls and 423 HCC patients were recruited for this case cohort study. According to our analysis of HCC patients, we found significant differences in age (*p* < 0.001) and alcohol consumption (*p* < 0.001) between HCC patients and the healthy group (Table 1).

To reduce possible confounding by several environmental factors, AORs and their corresponding 95% CIs were estimated by multivariate logistic regression models, after controlling for risks associated with age and alcohol consumption use. The genotype distributions and the associations between HCC and *MUC6* SNPs are presented in Table 2. The alleles with the highest frequency of distribution in *MUC6* rs61869016, rs6597947, and rs7481521 were homozygous T/T, homozygous C/C, and homozygous C/C, respectively, in HCC patients and controls. After adjusting for variables, individuals with rs61869016 C/C showed a 0.571-fold (95% CI: 0.380-0.858) lower risk of HCC. Individuals with the rs6597947 and rs7481521 polymorphisms showed no reduction in HCC risk compared to wildtype individuals.

In addition, the effect of the polymorphic genotypes of *MUC6* rs61869016 and rs7481521 on the clinical status of HCC was investigated (Tables 3 and 4). The results showed that patients with the C/C genotype of the rs61869016 SNP (OR = 3.515, 95% CI: 1.040-11.878, *p* = 0.043) and the T/T genotype of the rs7481521 SNP (OR = 4.582, 95% CI: 1.061-19.778, *p* = 0.041) had a higher Child-Pugh score (B or C) compared to other genotypes, suggesting poor survival in patients with chronic liver disease.

Moreover, we analyzed the levels of AFP, AST, and ALT, common clinicopathological markers of HCC associated with *MUC6* genotype frequency, to see how they related to the progression of clinical status in HCC patients. The homozygous genotype for the polymorphic allele of rs6597947 (C/A + A/A) had a significantly higher AST/ALT ratio compared with the C/C genotype in patients with HCC (Table 5).

## Discussion

SNPs are single-nucleotide variants that occur at the DNA level in each human cell. Associated with environmental factors, SNPs can not only mimic the diversity of the human phenotype, but also indicate susceptibility to a variety of diseases, including cancer 23. The association between SNPs and HCC has been tested in case-control and prospective cohort studies, which are based on hypothesis-driven, hypothetical genetic studies. For example, many of the changes affecting inflammatory pathways, oxidative stress, iron metabolism, or DNA repair mechanisms in hepatitis patients have been associated with the development of liver cancer 24.

Although the importance of MUC6 in cancer is well recognized, its exact role in tumorigenesis remains a controversial topic, as both oncogenic and inhibitory effects have been demonstrated 16, 25, 26. For example, *MUC6* is highly expressed at early noninvasive stages of pancreatic tumor progression and then suppressed or lost at invasive stages 27, 28. The *MUC6* SNP rs7481521 had a significant association with a decreased risk for homozygous carriers and a significant dose-response relation with the number of alleles in chronic atrophic gastritis patients 20. However, the correlation between *MUC6* polymorphisms and risk factors in HCC has not yet been clarified. The results of this study clarify the role of *MUC6* SNPs in HCC susceptibility and other clinicopathological conditions.

Taiwan is a region where viral hepatitis is very common; thus, if the AST/ALT ratio is high in Taiwan, the most likely cause is chronic hepatitis B, hepatitis C, or fatty liver disease 29-32. In our results, we observed that *MUC6* SNP rs6597947 was correlated with a significantly higher AST/ALT ratio in HCC patients. Moreover, the ALT value exceeded the normal value of 40 IU/L in patients with *MUC6* SNP rs61869016 and rs7481521, indicating liver damage (Table 5). As shown in Table 4, patients with the C/C genotype of SNP rs61869016 and the T/T genotype of SNP rs7481521 had higher Child-Pugh scores (B or C), suggesting poor survival in patients with chronic liver disease. In chronic viral liver disease, including chronic viral hepatitis, chronic alcoholism, and nonalcoholic fatty liver disease, an elevated AST/ALT ratio can be interpreted as a predictor for assessing long-term complications such as fibrosis and cirrhosis. In recent years, chronic liver inflammation has been the subject of intense research and is thought to have the potential to progress to liver cancer 29. Interestingly, after adjusting for variables, individuals with rs61869016 C/C showed a lower risk of HCC (Table 2). Unfortunately, the sample size of MUC6 polymorphism for C/C at rs61869016 is 37 patients and we cannot provide the prognosis data for MUC6 polymorphism for CC at rs61869016 in this current study. However, the detailed mechanisms of *MUC6* SNPs in HCC require future elucidation.

## Conclusions

In conclusion, our findings suggest that genetic variations in *MUC6* may help to predict cancer susceptibility and hepatitis in HCC. This study provides new information about the relationship between *MUC6* polymorphisms and the clinical pathology of HCC in the Taiwanese population.

## Figures and Tables

**Table 1:** Demographical characteristics of 1197 controls and 423 patients with HCC

Variable	Controls (*N* = 1197)	Patients (*N* = 423)	*p*-value
**Age (yrs)**	Mean ± SD	Mean ± SD	
	59.4 ± 7.1	63.7 ± 11.2	*p* < 0.001*
**Gender**			
Male	838 (70.0%)	298 (70.4%)	
Female	359 (30.0%)	125 (29.6%)	*p* = 0.865
**Cigarette smoking**			
No	727 (60.7%)	259 (61.2%)	
Yes	470 (39.3%)	164 (38.8%)	*p* = 0.858
**Alcohol drinking**			
No	1028 (85.9%)	279 (66.0%)	
Yes	169 (14.1%)	144 (34.0%)	*p* < 0.001*
**HBsAg**			
Negative		247 (58.4%)	
Positive		176 (41.6%)	
**Anti-HCV**			
Negative		241 (57.0%)	
Positive		182 (43.0%)	
**Stage**			
I + II		305 (72.1%)	
III + IV		118 (27.9%)	
**Tumor T status**			
T1 + T2		311 (73.5%)	
T3 + T4		112 (26.5%)	
**Lymph node status**			
N0		412 (97.4%)	
N1 + N2 + N3		11 (2.6%)	
**Metastasis**			
M0		400 (94.6%)	
M1		23 (5.4%)	
**Vascular invasion**			
No		359 (84.9%)	
Yes		64 (15.1%)	
**Child-Pugh score**			
A		362 (85.6%)	
B or C		61 (14.4%)	
**Liver cirrhosis**			
Negative		68 (16.1%)	
Positive		355 (83.9%)	

Mann-Whitney U test or Fisher's exact test was used between healthy controls and patients with HCC. * *p* < 0.05 was considered statistically significant.

**Table 2:** Genotype and allele frequency of *MUC6* single-nucleotide polymorphism (SNPs) in HCC patients and normal controls

Variable	Controls (*N* = 1197) (%)	Patients (*N* = 423) (%)	OR (95% CI)	AOR (95% CI)^a^
**rs61869016**				
TT	497 (41.5%)	191 (45.1%)	1.000 (reference)	1.000 (reference)
TC	541 (45.2%)	195 (46.1%)	0.938 (0.742-1.185)	0.912 (0.717-1.160)
CC	159 (13.3%)	37 (8.8%)	**0.606 (0.408-0.899)^b^**	**0.571 (0.380-0.858)^c^**
TC + CC	700 (58.5%)	232 (54.9%)	0.862 (0.690-1.078)	0.834 (0.662-1.050)
**rs6597947**				
CC	644 (53.8%)	229 (54.1%)	1.000 (reference)	1.000 (reference)
CA	460 (38.4%)	165 (39.0%)	1.009 (0.799-1.274)	1.013 (0.797-1.288)
AA	93 (7.8%)	29 (6.9%)	0.877 (0.563-1.366)	0.931 (0.591-1.466)
CA + AA	553 (46.2%)	194 (45.9%)	0.987 (0.790-1.232)	0.999 (0.795-1.257)
**rs7481521**				
CC	605 (50.5%)	204 (48.2%)	1.000 (reference)	1.000 (reference)
CT	486 (40.6%)	192 (45.4%)	1.172 (0.931-1.475)	1.137 (0.896-1.442)
TT	106 (8.9%)	27 (6.4%)	0.756 (0.481-1.187)	0.717 (0.451-1.141)
CT + TT	592 (49.5%)	219 (51.8%)	1.097 (0.879-1.370)	1.061 (0.844-1.333)

^a^ Adjusted for the effects of age and alcohol drinking; ^b^ *p* = 0.013; ^c^ *p* = 0.001.

**Table 3:** Odds ratio (OR) and 95% confidence interval (CI) of clinical status and *MUC6* rs61869016 genotypic frequencies in HCC patients among smokers

Variable			OR (95% CI)	*p*-value
**Clinical Stage**				
**rs61869016**	Stage I + II (*n* = 118) (%)	Stage III + IV (*n* = 46) (%)		
TT	44 (37.3%)	23 (50.0%)	1.00	
TC	60 (50.8%)	20 (43.5%)	0.638 (0.312-1.303)	*p* = 0.217
CC	14 (11.9%)	3 (6.5%)	0.410 (0.107-1.574)	*p* = 0.194
**Tumor size**				
**rs61869016**	≤T2 (*n* = 118) (%)	>T2 (*n* = 46) (%)		
TT	43 (36.4%)	24 (52.2%)	1.00	
TC	61 (51.7%)	19 (41.3%)	0.588 (0.272-1.143)	*p* = 0.111
CC	14 (11.9%)	3 (6.5%)	0.384 (0.100-1.471)	*p* = 0.163
**Lymph node metastasis**				
**rs61869016**	No (*n* = 160) (%)	Yes (*n* = 4) (%)		
TT	66 (41.3%)	1 (25.0%)	1.00	
TC	77 (48.1%)	3 (75.0%)	2.571 (0.261-25.315)	*p* = 0.418
CC	17 (10.6%)	0 (0.0%)	-	-
**Distant metastasis**				
**rs61869016**	M0 (n = 156) (%)	M1 (n = 8) (%)		
TT	62 (39.7%)	5 (62.5%)	1.00	
TC	77 (49.4%)	3 (37.5%)	0.483 (0.111-2.101)	*p* = 0.332
CC	17 (10.9%)	0 (0.0%)	-	-
**Vascular invasion**				
**rs61869016**	No (*n* = 138) (%)	Yes (*n* = 26) (%)		
TT	59 (42.8%)	8 (30.8%)	1.00	
TC	66 (47.8%)	14 (53.8%)	1.564 (0.613-3.993)	*p* = 0.349
CC	13 (9.4%)	4 (15.4%)	2.269 (0.593-8.684)	*p* = 0.231
**Child-Pugh score**				
**rs61869016**	A (*n* = 141) (%)	B or C (*n* = 23) (%)		
TT	58 (41.1%)	9 (39.1%)	1.00	
TC	72 (51.1%)	8 (34.8%)	0.716 (0.260-1.972)	*p* = 0.518
CC	11 (7.8%)	6 (26.1%)	**3.515 (1.040-11.878)**	*p* = 0.043^*^
**HBsAg**				
**rs61869016**	Negative (*n* = 93) (%)	Positive (*n* = 71) (%)		
TT	39 (41.9%)	28 (39.4%)	1.00	
TC	43 (46.3%)	37 (52.1%)	1.199 (0.623-2.307)	*p* = 0.588
CC	11 (11.8%)	6 (8.5%)	0.760 (0.251-2.298)	*p* = 0.627
**Anti-HCV**				
**rs61869016**	Negative (*n* = 92) (%)	Positive (*n* = 72) (%)		
TT	36 (39.1%)	31 (43.1%)	1.00	
TC	45 (48.9%)	35 (48.6%)	0.903 (0.470-1.734)	*p* = 0.760
CC	11 (12.0%)	6 (8.3%)	0.633 (0.210-1.912)	*p* = 0.418
**Liver cirrhosis**				
**rs61869016**	Negative (*n* = 26) (%)	Positive (*n* = 138) (%)		
TT	13 (50.0%)	54 (39.1%)	1.00	
TC	11 (42.3%)	69 (50.0%)	1.510 (0.627-3.635)	*p* = 0.358
CC	2 (7.7%)	15 (10.9%)	1.806 (0.366-8.897)	*p* = 0.468

The ORs analyzed by their 95% CIs were estimated by logistic regression models; >T2: multiple tumors more than 5 cm or tumor involving a major branch of the portal or hepatic vein(s); * *p* < 0.05 was considered statistically significant.

**Table 4:** Odds ratio (OR) and 95% confidence interval (CI) of clinical status and *MUC6* rs7481521 genotypic frequencies in HCC patients among smokers

Variable			OR (95% CI)	*p*-value
**Clinical Stage**				
**rs7481521**	Stage I + II (*n* = 118) (%)	Stage III + IV (*n* = 46) (%)		
CC	51 (43.2%)	23 (50.0%)	1.00	
CT	61 (51.7%)	20 (43.5%)	0.727 (0.359-1.472)	*p* = 0.376
TT	6 (5.1%)	3 (6.5%)	1.109 (0.255-4.826)	*p* = 0.891
**Tumor size**				
**rs7481521**	≤T2 (*n* = 118) (%)	>T2 (*n* = 46) (%)		
CC	52 (44.1%)	22 (47.8%)	1.00	
CT	60 (50.8%)	21 (45.6%)	0.827 (0.409-1.672)	*p* = 0.598
TT	6 (5.1%)	3 (6.6%)	1.182 (0.271-5.155)	*p* = 0.824
**Lymph node metastasis**				
**rs7481521**	No (*n* = 160) (%)	Yes (*n* = 4) (%)		
CC	73 (45.6%)	1 (25.0%)	1.00	
CT	78 (48.8%)	3 (75.0%)	2.808 (0.286-27.603)	*p* = 0.376
TT	9 (5.6%)	0 (0.0%)	-	-
**Distant metastasis**				
**rs7481521**	M0 (*n* = 156) (%)	M1 (*n* = 8) (%)		
CC	70 (44.9%)	4 (50.0%)	1.00	
CT	77 (49.3%)	4 (50.0%)	0.909 (0.219-3.773)	*p* = 0.896
TT	9 (5.8%)	0 (0.0%)	-	-
**Vascular invasion**				
**rs7481521**	No (*n* = 138) (%)	Yes (*n* = 26) (%)		
CC	66 (47.8%)	8 (30.8%)	1.00	
CT	65 (47.1%)	16 (61.5%)	2.031 (0.813-5.071)	*p* = 0.129
TT	7 (5.1%)	2 (7.7%)	2.357 (0.416-13.353)	*p* = 0.333
**Child-Pugh score**				
**rs7481521**	A (*n* = 141) (%)	B or C (*n* = 23) (%)		
CC	63 (44.7%)	11 (47.8%)	1.00	
CT	73 (51.8%)	8 (34.8%)	0.628 (0.238-1.657)	*p* = 0.347
TT	5 (3.5%)	4 (17.4%)	**4.582 (1.061-19.778)**	*p* = 0.041^*^
**HBsAg**				
**rs7481521**	Negative (*n* = 93) (%)	Positive (*n* = 71) (%)		
CC	41 (44.1%)	33 (46.5%)	1.00	
CT	47 (50.5%)	34 (47.9%)	0.899 (0.476-1.698)	*p* = 0.742
TT	5 (5.4%)	4 (5.6%)	0.994 (0.247-4.000)	*p* = 0.993
**Anti-HCV**				
**rs7481521**	Negative (*n* = 92) (%)	Positive (*n* = 72) (%)		
CC	45 (48.9%)	29 (40.3%)	1.00	
CT	40 (43.5)	41 (56.9%)	1.591 (0.840-3.012)	*p* = 0.154
TT	7 (7.6%)	2 (2.8%)	0.443 (0.086-2.284)	*p* = 0.331
**Liver cirrhosis**				
**rs7481521**	Negative (*n* = 26) (%)	Positive (*n* = 138) (%)		
CC	13 (50.0%)	61 (44.2%)	1.00	
CT	12 (46.2%)	69 (50.0%)	1.225 (0.520-2.887)	*p* = 0.642
TT	1 (3.8%)	8 (5.8%)	1.705 (0.196-14.833)	*p* = 0.629

The ORs analyzed by their 95% CIs were estimated by logistic regression models; >T2: multiple tumors more than 5 cm or tumor involving a major branch of the portal or hepatic vein(s); * *p* < 0.05 was considered statistically significant.

**Table 5:** Association of *MUC6* genotypic frequencies with the HCC laboratory findings

Characteristic	α-Fetoprotein^a^ (ng/mL)	AST^a^ (IU/L)	ALT^a^ (IU/L)	AST/ALT^a^ ratio
**rs61869016**				
TT	996.4 ± 364.4	44.8 ± 5.3	42.0 ± 4.5	1.23 ± 0.04
TC + CC	990.8 ± 325.9	42.8 ± 3.7	79.7 ± 39.4	1.18 ± 0.02
*p*-value	0.991	0.761	0.342	0.248
*p*-value^b^	0.935	0.779	0.398	0.214
**rs6597947**				
CC	1084.0 ± 362.2	41.4 ± 2.7	85.5 ± 42.1	1.15 ± 0.02
CA + AA	886.9 ± 314.3	46.3 ± 5.9	38.2 ± 3.7	1.26 ± 0.04
*p*-value	0.681	0.445	0.264	**0.004**
*p*-value^b^	0.705	0.424	0.297	**0.002**
**rs7481521**				
CC	784.8 ± 306.2	44.6 ± 5.0	43.7 ± 4.7	1.22 ± 0.03
CT + TT	1201.0 ± 377.4	42.7 ± 3.6	83.6 ± 45.2	1.18 ± 0.02
*p-*value	0.392	0.751	0.380	0.281
*p*-value^b^	0.471	0.784	0.354	0.275

The Mann-Whitney U test was used between two groups;^ a^ mean ± SE;^ b^ adjusted for age and alcohol drinking.
